# Quality of the parent-child interaction in young children with type 1 diabetes mellitus: study protocol

**DOI:** 10.1186/1471-2431-11-28

**Published:** 2011-04-14

**Authors:** Anke M Nieuwesteeg, Frans Pouwer, Hedwig JA van Bakel, Wilco HM Emons, Henk-Jan Aanstoot, Roelof Odink, Esther E Hartman

**Affiliations:** 1 Center of Research on Psychology in Somatic diseases [CoRPS], Department of Medical Psychology and Neuropsychology, Tilburg University, Tilburg, the Netherlands; 2 Department of Developmental Psychology, Tilburg University, Tilburg, the Netherlands; 3 Department of Methodology and Statistics, Tilburg University, Tilburg, the Netherlands; 4 Diabeter, Rotterdam, the Netherlands; 5 Department of Pediatrics, Catharina Hospital, Eindhoven, the Netherlands

## Abstract

**Background:**

In young children with type 1 diabetes mellitus (T1DM) parents have full responsibility for the diabetes-management of their child (e.g. blood glucose monitoring, and administering insulin). Behavioral tasks in childhood, such as developing autonomy, and oppositional behavior (e.g. refusing food) may interfere with the diabetes-management to achieve an optimal blood glucose control. Furthermore, higher blood glucose levels are related to more behavioral problems. So parents might need to negotiate with their child on the diabetes-management to avoid this direct negative effect. This interference, the negotiations, and the parent's responsibility for diabetes may negatively affect the quality of parent-child interaction. Nevertheless, there is little knowledge about the quality of interaction between parents and young children with T1DM, and the possible impact this may have on glycemic control and psychosocial functioning of the child. While widely used global parent-child interaction observational methods are available, there is a need for an observational tool specifically tailored to the interaction patterns of parents and children with T1DM. The main aim of this study is to construct a disease-specific observational method to assess diabetes-specific parent-child interaction. Additional aim is to explore whether the quality of parent-child interactions is associated with the glycemic control, and psychosocial functioning (resilience, behavioral problems, and quality of life).

**Methods/Design:**

First, we will examine which situations are most suitable for observing diabetes-specific interactions. Then, these situations will be video-taped in a pilot study (N = 15). Observed behaviors are described into rating scales, with each scale describing characteristics of parent-child interactional behaviors. Next, we apply the observational tool on a larger scale for further evaluation of the instrument (N = 120). The parents are asked twice (with two years in between) to fill out questionnaires about psychosocial functioning of their child with T1DM. Furthermore, glycemic control (HbA_1c_) will be obtained from their medical records.

**Discussion:**

A disease-specific observational tool will enable the detailed assessment of the quality of diabetes-specific parent-child interactions. The availability of such a tool will facilitate future (intervention) studies that will yield more knowledge about impact of parent-child interactions on psychosocial functioning, and glycemic control of children with T1DM.

## Background

Results of The Diabetes Control and Complications Trial (DCCT) have convincingly shown that keeping blood glucose levels close to normal levels avoids or delays the onset of long-term complications of diabetes [1]. When young children are diagnosed with type 1 diabetes mellitus (T1DM), parents get full responsibility for the diabetes-management of their child (e.g., blood glucose monitoring and administering insulin, regulation of food intake, and guarding the level of physical activity of their diabetic child). Normal and age appropriate behaviors that occur in the toddler and pre-school years (e.g. independence-seeking, refusing food, oppositional behavior) can interfere with the ability of parents to complete the tasks needed to achieve optimal blood glucose control [2]. This interference and the full responsibility of the parents may affect family functioning and parent-child interaction [3-5].

An overview by Anderson et al. [6] showed that when a child suffers from a chronic condition the parent-child relationship could be affected. Most studies described in this overview have shown negative effects of a medical condition on the quality of parent-child interactions, such as more conflict situations and less solution-directed communication, less cohesion, decreased medication adherence, and impaired functioning within the family [6]. For example, children with congenital heart disease reacted less responsive and their mothers appeared less sensitive than children and mothers in healthy families [7]. In families with a disturbed parent-child interaction, children with various somatic diseases showed more behavioral problems, but also more disease-related outcomes, such as an increased mean glycemic control (HbA_1c_) in adolescents with diabetes [8]. Higher glycosylated hemoglobin is associated with more behavioral problems in youth with type 1 diabetes [9]. Because of these possible behavioral problems, parents want to keep their child's blood glucose values as close to normal as possible, to avoid the direct negative effect on the behavior of their child. To achieve this, the parents might need to negotiate with their child on the diabetes management tasks, but diabetes treatment is non-negotiable. These negotiations could negatively affect the interaction between parent and child. Diabetes is a 24/7 disease, so struggles on treatment tasks are not comparable with other (chronic) diseases.

Because parents are responsible for the treatment of their young child with T1DM and the child is fully dependent on his or her parents, we expect that the quality of parent-child interaction significantly contributes to both the psychosocial development and the quality of life of these children.

Given the importance of the topic, it is surprising that studies examining the quality of the parent-child interaction in families with young children with T1DM are scarce. Furthermore, the small number of studies that are available has several shortcomings. For example, most studies examining the quality of parent-child interaction or related topics, have been conducted with older children with T1DM (> 8 years) [3,4,8,10,11], or used a wide age range (from 1 - 14 years [12,13]). As a result, specific knowledge of the quality of the parent child interaction of the youngest patient group is lacking, and no specific statements can be made about the quality of parent-child interaction in families with a young child with T1DM.

A second shortcoming is that in these studies self-report measures or semi-structured interviews were used [3,4,11-14]. In vivo observations and observational methods, however, appear more sensitive to subtle differences in family interactions [15]. These differences in interaction patterns may provide important implications for improving the quality of parent-child interactions during diabetes-management. Moreover, self-report measures and interviews reflect a subjective view from the perspective of parents, while by using observational methods, the interaction patterns can be assessed more objectively.

To our knowledge, there is only one research group [2,16-19], that has studied the quality of parent-child interaction in young children with T1DM using an observational method. However, the studies of this research group only focused on a single dimension of a disease-specific parent-child situation, namely behavioral problems during the meal, while in fact the combination of diabetes-specific actions and behaviors around mealtime will give a more complete illustration of the diabetes-specific interactions (i.e. blood glucose monitoring, carbohydrate counting, and administering insulin).

Moreover, the observations were performed with an observational method [20] in which the behaviors of parents and children during the meal were to be counted (e.g., how often the child was encouraged to keep eating). This has an important disadvantage. In behavior counting methods, where all behaviors are counted, applying nuances is difficult, while with the so-called "rating scales" specific behaviors can be grouped under broad categories. This way of coding observational data provides room to make many dimensions and nuances in behaviors. Moreover, the predictive value of global rating scales has proved to be more appropriate than just counting specific behaviors [15,21]. An additional advantage of rating scales is that it costs up to 5 times less time than counting all behaviors [15]. The use of rating scales in observational studies is not only time efficient but also gives a clinical picture which results in more specific implications for intervention purposes.

Another limitation of these studies is that generic parent-child interactions were not observed [2,16-19]. It might be that diabetes-specific parent-child interactions affects child behavior (such as gaining independence, stubbornness, oppositional behavior) and parental behavior (such as sensitivity, respecting autonomy, having fun together) [22] which might negatively affect the daily family life and the generic quality of the parent-child interaction.

The results of the cross-sectional studies of Patton et al. [2,16,17] showed that the behavior of the children and parents during mealtime were associated with the glycemic control (HbA_1c_), the more family malfunctioning, the higher the glycemic control (HbA_1c_). However, longitudinal studies are currently lacking in this area.

We expect that the way in which parents treating their child with T1DM during these often annoying, sometimes even invasive, yet unavoidable procedures can affect blood glucose control (HbA_1c_) and psychosocial functioning. For example, if the mother responds anxiously when she has to monitor her child's blood glucose, the child may start to cry. If the mother then decides to postpone the finger prick this could lead to a hyperglycemia. Some parents may also have strong worries about future complications or hypoglycemic events. Because of these concerns, parents may decide monitor the child's blood glucose 15 times a day, three times per night, once at 23:00, and even once at 2:00 pm and once at 5:00 pm. These examples of non-constructive interaction patterns can be physically and emotionally stressful for the child (and parents), which might disturb the balance between effective treatment and optimal quality of life.

Because diabetes-related behaviors are usually consolidated in the first years post diagnosis [12,23], interventions should start as early as possible. The combination of observing both generic and disease-specific interactions will identify interactional patterns to evaluate future behavioral interventions, with the aim of learning more effective parent-child interactions to optimize the glycemic control and psychosocial functioning of young children with T1DM as early as possible to prevent future problems.

In the past decades, several global rating scales have been developed for assessing different aspects of the quality of parent-child interactions, e.g., the Emotional Availability Scales (EAS) [24] and the scales developed by Erickson, Sroufe and Egeland [25]. These measures were designed to cover different aspects of parent-child interaction irrespective of an underlying disease. Disease-specific measures could assess interactions between parents and children at a disease-specific level (e.g., during administering insulin, mealtime behavior). Disease-specific instruments are expected to be more responsive to small changes that are important to clinicians or patients [26]. However, such a disease-specific observational measure is not available for children with T1DM. The main aim of the present study is to develop a disease-specific observational method, including a scoring system, to assess diabetes-specific parent-child interaction and to test the initial and preliminary psychometric properties of the pilot version of the instrument. Additional aim is to explore whether quality of parent-child interactions is associated with the glycemic control, child behaviors, and quality of life of children with T1DM.

## Methods/Design

### Developing the OKI-DO observation method

**Figure 1:**
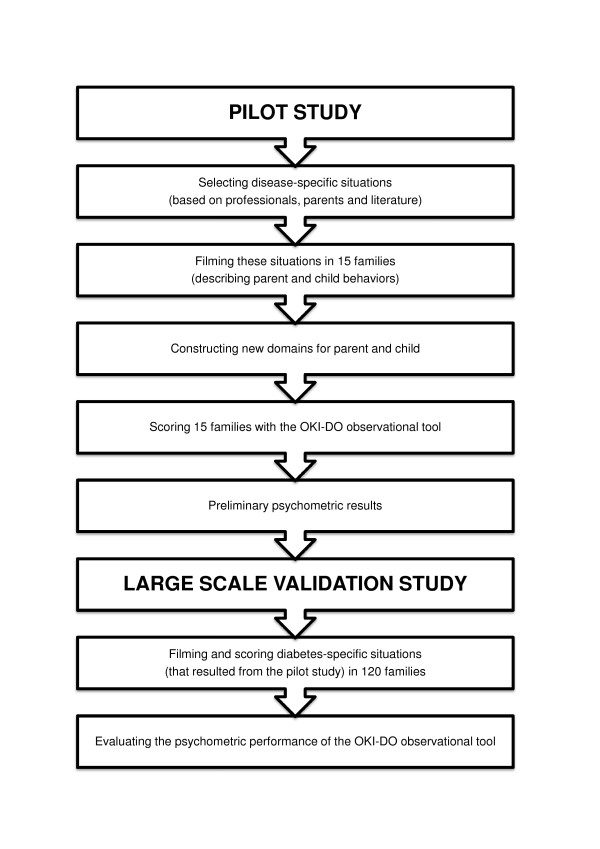
**Development of the observational method to assess the diabetes-specific quality of the parent-child interaction: This figure shows the steps in developing and validating the OKI-DO method**.

The development of the OKI-DO observation method (OKI-DO, Ouder-Kind Interactie Diabetes Onderzoek, which means: Parent-Child Interaction Diabetes Research) proceeds in two steps: (1) a small scale pilot study, and (2) a large scale validation study (see Figure 1).

1. In the small scale pilot study we will develop the OKI-DO observation method in three steps: (a) defining relevant disease-specific situations; (b) development of a child and parent behavior list and making the observation method; (c) pilot test of the observational method. These three steps will be described below.

a) The first step in the development of the OKI-DO method is to select disease-specific situations (e.g., administering insulin) that are most relevant for parents and children with T1DM. The selection of the situations will be based on theory, literature, and interviews with pediatricians, diabetes-nurses and parents.

b) In the second step we will identify which disease-specific parent-child interactions play a role in diabetes-specific situations and how these interactions can be used to make our observation instrument. Therefore, the selected disease-specific situations will be videotaped during a home-visit in 15 families. Parent behaviors (e.g., comforting or distracting during the administering of insulin) and child behaviors (e.g., crying or accepting) will be described by a trained psychologist (HJAvB) and a research assistant (AMN). All parent and child behaviors will be described and will be specified from the observed parent and child behaviors by expert opinions, psychological theory, and the generic domains of parent-child interaction [24,25]. The final result will be a list of rating scales, where each scale will be described by characteristics of parent-child interaction behaviors. We intend to develop complete descriptions of behaviors. An example of a pediatric diabetes-specific parent scale during administering insulin could be "parents supportive presence during administering insulin", which can be rated on a 7-point Likert scale varying from 1: *"Mother (or father) completely fails to be supportive to the child, being unavailable or being hostile toward the child when the child shows need of some support during this situation" *to 7: *"Mother (or father) skillfully provides support throughout the session. From the beginning she/he is confident that the child is capable of enduring this situation. If the child is having difficulty, she/he finds ways to calm the child and encourages positive behavior. Mother/father is not only emotionally supportive but continuously reinforces the child for good behavior (e.g., being compliant)"*. In the same manner other scales could be constructed, such as *"Parents respect for child's autonomy during administering insulin" *and *"Mothers/Fathers quality of instructions during administering insulin"*. Scales of child behaviors might become for example *"Avoidance of the parent during administering insulin" *and *"Compliant/cooperative during administering insulin*".

c) The third and final step is to test the feasibility of the behavioral rating scale that has been developed in the previous two steps. The observational tool will be tested in a small-scale pilot study (N = 15).

2. Second, we will apply the observational tool in a large scale study to collect data for further evaluation of the reliability and the validity of the instrument (N≈120). In addition, we will explore whether the quality of the parent-child interactions is associated with the glycemic control and psychosocial functioning (resilience, behavioral problems, and quality of life). Therefore, the parents are asked twice (with two years in between) to fill out questionnaires about psychosocial functioning of their child with T1DM. Glycemic control (HbA_1c_) will be obtained from their medical records.

### Procedure and participants

For this study, infants, toddlers and (pre)school children (aged 0-7 years) with T1DM and their parents will be recruited from several hospitals/institutions in the middle and southern part of the Netherlands (St. Elisabeth Hospital Tilburg, TweeSteden Hospital Tilburg, Catharina Hospital Eindhoven, St. Anna Hospital Geldrop, Bernhoven Hospital Veghel/Oss, Jeroen Bosch Hospital Den Bosch, Elkerliek Hospital Helmond, and Diabeter Rotterdam). We expect to approach about 175 young patients with type 1 diabetes mellitus and their parents. Assuming a response rate of 70%, we expect to collect data on 122 dyads in the present study. Families who agree to participate will be visited in their homes. After making an appointment, a set of questionnaires will be sent to the parents. The child and his or her parent(s) will be videotaped during 1. a free play task (e.g., playing with clay or making a puzzle), and 2. a number of disease-specific situations (e.g., administering insulin and mealtime behavior). The free play task will be rated with the scales developed by Erickson, Sroufe and Egeland [25], and the disease-specific situations will be rated with the OKI-DO instrument. At the end of the visit the questionnaires, which were filled out by the parents, will be collected. Glycemic control (HbA_1c_) will be obtained from the medical record of the children.

### Ethical considerations

The study design has been approved by the medical ethical committee of St. Elisabeth Hospital Tilburg (date: 25-05-2010). All parents/guardians are provided with written information about the study and are asked to give written informed consent prior to filming.

### Study measures

#### Sociodemographic and clinical data

Parents will be asked to fill in a questionnaire with demographic background information (gender, date of birth, living situation, siblings, school, religion, land of birth, marital status, and education level) and clinical data (time since diagnose, disease-duration, treatment regimen, i.e., insulin pump, insulin injections, number of injections per day, number of glucose monitoring per day, number of hypoglycemic events for the past 3 months, hospital/institution, and hospitalization).

#### Quality of the parent-child interaction

In the large scale validation study, the disease-specific quality of the parent-child interaction will be measured with the observation method that has been developed in the small scale pilot-study (OKI-DO instrument). During a home-visit the child and his or her parents will be videotaped during 2 or 3 disease-specific situations (e.g., administering insulin). Their behaviors will be scored using the OKI-DO rating scales.

To assess generic quality of the parent-child interaction, the child and his or her parents are videotaped during a free play situation (e.g., playing with clay or making a puzzle; toys appropriate to the age and interests of the child) in the home situation, which will be rated by the scales developed by Erickson et al. [25]. These scales asses different domains of parent behavior and child behavior. Parental behavior includes the domains: supportive presence or the provision of emotional support, respect for the child's autonomy or non-intrusiveness, structure and limit setting, quality of instructions, and hostility. Child domains include negativity or anger, dislike or hostility, avoidance of interaction with the parent and compliance with suggestions and directions given by the parent. Each domain is scored on a scale ranging from 1 (low quality of parent-child interaction) through 7 (high quality of parent-child interaction).

#### Psychosocial functioning and quality of life of the children

Generic quality of life will be measured with the TNO-AZL Preschool Quality Of Life questionnaire or TAPQOL [27] in children in the age of 1 through 5 years of age and the TNO-AZL Child Quality Of Life questionnaire (TACQOL) [28] in children of 6 years and older. These questionnaires measure parent's perceptions of health-related quality of life in (preschool) children. The TAPQOL is a multidimensional instrument with 43 items divided into 12 scales covering the following aspects: sleeping problems, appetite, lung problems, stomach problems, skin problems, motor functioning, social functioning, problem behavior, communication, anxiety, positive mood, and liveliness. The TACQOL is a multidimensional instrument with 63 items constituting 7 scales covering aspects of quality of life: five health-related scales: pain and symptoms, motor function, autonomy, cognitive functioning, interaction with parents and peers, and two scales that represent positive or negative emotions of a patient: experience of positive emotions and experience of negative emotions. In each of the health-related functioning scales, the parent can indicate to what extent specific problems occurred in the past few weeks, with three response categories: 'never', 'sometimes', and 'often'. If a problem occurs, the parents are asked how the child is feeling: '(very) good', 'not so good', 'pretty bad' and 'bad'. For each item, the two answers are combined into a single item score ranging from 0 to 4 ('never' 4 and 'sometimes' or 'often' combined with '(very) good' 3, 'not so good' 2, 'pretty bad' 1, and 'bad' 0). With the emotion scales, the parents indicate on a Likert scale or a certain emotion in their child has appeared in the last few weeks ('never', 'sometimes', 'often'). Item scores for the two emotion scales run from 0 to 2. In all TAPQOL and TACQOL scales, higher scores will correspond to a better quality of life. Reference values are given in the manual.

The diabetes-specific quality of life is measured with a child self-report questionnaire, composed by the Hvidøre Study Group. This questionnaire has been modified with permission of the authors so that the parents can complete the questionnaire for their child (proxy-report). The scale comprises 19 items about feelings of the child in relation with their diabetes (e.g. about administer insulin (injecting or pump) my child feels ...), health (e.g. my child felt fit and healthy), leisure time (e.g. my child had enough time to play) and school (e.g. school/nursery/daycare went well). The items are rated on a 5-point Likert scale (e.g. 'very happy', 'happy', 'neutral', 'sad', and 'very sad'). Scores are coded so that a higher score corresponds to a better quality of life. Currently, this questionnaire is widely used, and will be validated, by the Hvidøre Study Group.

The degree of psychosocial problems will be measured using the Strengths and Difficulties Questionnaire (SDQ) [29]. The SDQ is a brief behavioral screening questionnaire and measures the presence of psychosocial problems, the strengths of the child and the influence of psychosocial problems in daily functioning. The SDQ is suitable for children of 3 years and older. The questionnaire contains 25 items, covering the following five domains: conduct symptoms, hyperactivity/inattention, emotional problems, peer relationship problems, and pro-social behavior. The 25 items were formulated on the basis of propositions (e.g., "Considerate of other people's feelings") and relate to the past 6 months. Some propositions are oppositely formulated, such as "Thinks before acting out." Therefore, the subscales have a bipolar character, that is, a low score not only means that there are no problems, but also that there are one or more strengths [30]. Research showed that the SDQ is a reliable and valid questionnaire [31].

To measure potential behavioral problems of children less than three years, the Child Behavior Check List (CBCL) [32] is used. The CBCL 1.5-5 consists of 99 items that are categorized into seven scales, including emotionally reactive, anxious/depressed, somatic complaints, withdrawal, sleep problems, attention problems, and aggressive behavior. The items can be further summarized into internalizing problems and externalizing problems or a total problem score (adding up all items).

### Data analyses

The aim of this study is to develop an observational method that provides a standardized procedure to collect information on parent-child interactions in young children by means of direct observations from videotaped diabetes-specific situations. Before the observational method can be used for substantive research purposes, it has to be empirically examined to what extent the observation method provides reliable and valid information on diabetes-specific parent-child interactions. In this study, we will first test the observational tool in a small-scale pilot study (N = 15). Second, we will apply the observational tool on a larger scale to collect data for further evaluation of the reliability and the validity of the instrument (N≈120).

#### 1. Small scale Pilot Test

In the pilot study, three raters will score parent-child interactions for 15 pediatric diabetes patients in videotaped home situations. Two of the project leaders (EEH, HJAvB) and a research assistant (AMN) will independently rate the video-tapes. One of the raters (HJAvB) is an expert on parent-child interactions and has a lot of experience with various rating scales in different populations. After the raters have rated the 15 patients, a debriefing questionnaire will be administered in which the raters are asked to appraise the feasibility of the observational tool (are the instructions clear; do instructions need further explications) and to comment on usefulness and face validity of the scales and its constituent indicators. In addition, the inter-rater reliability will be determined by means of inter-rater reliability indices (e.g., Intra Class Correlation Coefficients, ICC). Based on qualitative data from the rater's feedback and the statistical result, the observational tool may be refined if necessary (e.g., making the instructions more specific or explicit). If major revisions are needed, the pilot test will be repeated using same groups of raters. The pilot study must result in an experimental version of the observational tool that is deemed applicable, valid, and appropriate for diabetes-specific situations by an expert panel and the raters.

#### 2. Large Scale Validation Study

In the validation study, data of 120 (pre-)school children with T1DM and their parents from several hospitals/institutions will be analyzed. The reliability of the OKI-DO observation method is defined by the degree of inter-rater reliability and the reliability of average ratings across raters, inter-rater agreement, and will be assessed using weighted kappa [33] and intra-class correlation coefficients [34]. Reliability values ≥ 0.70 are generally accepted as adequate for scientific research. The validity of the OKI-DO observation method is assessed in different ways.

First, we will evaluate the face validity of the instrument. We will describe all observed parent and child behaviors as complete and precisely as possible to make sure the OKI-DO observation method will measure the quality of interaction between parent and child.

Second, we will examine construct validity by testing predictions on the relations with other parent-child interaction scales. If these predictions are supported by the data, we have supportive evidence for the construct validity of the instrument [35]. In particular, we will test associations of scores obtained with the OKI-DO method with scores from related observations tools assessing global parent-child interaction [25].

We expect that global interaction scales [25] and the OKI-DO scales will correlate substantially (Spearman's rho > 0.40), whereas conceptually unrelated scales (like 'negativity' and 'enthusiasm') will correlate less than 0.20. We will also compare the correlation of a few scales of the included questionnaires with the OKI-DO method. One example includes a comparison of the scale 'aggressive behavior' from the CBCL with a scale from the OKI-DO method that measures something like 'hostility of the child'.

Third, validity will be assessed with the method of known-group comparisons [36] to evaluate the extent to which the observational tool will be able to discriminate between subgroups of patients differing in time since diagnosis and glycemic control ('good' versus 'bad' HbA_1c_) and subgroups of parents differing in gender (father/mother). We believe that children who are recently diagnosed with T1DM and children who have a 'bad' glycemic control will have a poorer quality of parent-child interaction than children who are diagnosed with T1DM for a long time and children with a 'good' glycemic control. We also believe that mothers and fathers will differ in the quality of parent-child interaction, because mothers mostly have a closer relationship with their child [37].

#### 3. Substantive analysis on relation between parent-child interactions, psychosocial functioning and glycemic control

Third, we will study the relationship between parent-child interactions as measured by our observational tool and with the generic scales developed by Erickson et al. [25] with (changes in) the glycemic control (HbA_1c_), and psychosocial functioning (resilience, behavioral problems, and quality of life) of the children. These relationships will be tested with multiple regression analyses.

### Sample size/power analysis

We expect to include about 120 families (parent-child pairs) in this study, in which all 120 pairs are judged by three raters. To justify the sample size, we did several checks on the precision with which inter-rater reliability can be estimated and the power of testing hypotheses on correlations.

For assessing the inter-rater reliability (i.e., consistency among fixed raters), we use the ICC(3) [34]. Using confidence intervals reported in Shrout and Fleiss [34] we can assess the precision of ICC(3) estimates. For 120 observations and three raters, a 90% CI for an ICC(3) of .70 ranges from .63 to .76. Furthermore, with 120 observations and three raters, we have 80% power to find an inter-rater reliability of .74 or higher when tested against .70 (two-tailed test; *α *= .05). For the substantive analyses (i.e., testing relations of ratings with other variables), we will use mean ratings across the three raters per parent-child dyad. With 120 observations and three raters, a 90% confidence interval for a reliability of .80 for the mean ratings runs from .74 to .85.

To estimate the inter-rater agreement, which reveals important information about the feasibility of the instrument as an observational tool in clinical practice, we examine pair wise inter-rater agreement using kappa (i.e., treating observations as nominal ratings) and weighted kappa (treating observations as ordinal ratings) [38]. Using results from Hanley [39], the expected standard error of kappa in a sample of 120 will be in between .07 (for kappa 0.3) and .05 (for kappa = .8). For example, for a kappa value of .6, the 90% confidence interval ranges from .48 to .72. This approximation is based on 4-point ratings. However, as we will use seven-category ratings, the true standard error will likely be smaller and our kappa estimates will be more precise than indicated here. For valid application of weighted kappa for ratings on 7-point Likert scales, we used results from Cicchetti [40], who showed that the required minimum sample size is given by 2 × 7^2 ^= 98.

Power with respect to correlations: With Gpower 3.0 we calculated that finding population correlations of .4 or higher with at least a power of .9 (two-tailed *t*-test; *α *= .05) minimum samples sizes of 58 are needed. Power analyses showed that with regression analyses there will be sufficient power to find an explained variance of 15 percent or more (*F*-test, *α *= .05). For example, for 4 predictors and *N *= 120, the power for finding effect sizes of magnitude *R*^2 ^≥.15 is .93 (calculated with Gpower 3.0).

In sum, power and precision analysis showed that 120 dyads should be suffice to accurately estimate inter-rate reliability and agreement, and in sufficient power to find correlations between measures of parent-child interactions and the outcome measures.

### Possible results/relevance

The availability of a disease-specific observational instrument will enable the detailed assessment of the quality of the disease-specific parent-child interaction. More specifically, the instrument can be used to conduct studies that can help to determine which parent-child interaction-patterns are associated with specific diabetes outcomes, such as self care and glycemic control (HbA_1c_). Results will also show whether, and how, quality of the disease-specific interaction during these procedures is related to generic interaction between children and their parents. Further, we expect that the quality of the parent-child interaction will appear to affect glycemic control (HbA_1c_), child behaviors and quality of life. In future research, the OKI-DO observational method can be used as an evaluative tool to measure changes in the diabetes-specific interaction patterns across time, e.g., as a result of interventions based on the outcomes of this study.

## Discussion

The purpose of this study is to develop an observational tool to assess the disease-specific parent-child interactions in families with young children with T1DM. We will test initial and preliminary psychometrics of the pilot version of the OKI-DO instrument and then test the psychometric performance of the OKI-DO instrument in the large scale validation study. Sound observational methods enable scientists and clinical practitioners to compare objective behaviors at different time points and to evaluate interventions. When the OKI-DO method appears to be psychometrically sound, it will be used in future studies. The observational tool will be made available for (international) use by other research groups as well (English version).

To minimize observational bias, we will observe parent-child interactions in the home situation, where it is more likely that routine and daily patterns of interaction will be revealed compared to clinical settings [41].

Results of the present disease-specific observation study will identify behaviors that should be targets for future behavioral interventions for young children with T1DM and their parents. For health care providers to be able to inform, treat and refer patients and their parents to needed types of care, it is important to have insight into the parent (mothers and father) -child interactions in families with these young patients. Moreover, as diabetes-related family behaviors seem to be established in the early years post-diagnosis, interventions should start as early as possible. Ideally, future interventions should increase the strengths and decrease the weaknesses of global and disease-specific interaction patterns in families with children with T1DM, preferably in early childhood. Or, as an English proverb says "What's learnt in the cradle lasts till the tomb".

## Competing interests

The authors declare that they have no competing interests.

## Authors' contributions

Present investigation was developed by EEH, FP and HJAvB of The Center of Research on Psychology in Somatic diseases (CoRPS) and the department of Developmental Psychology at Tilburg University. HJA and RO are the study-coordinators of respectively Diabeter and Kidz&Ko, which are collaborating institutions. WHME is a methodologist at Tilburg University and assisted with the statistics and power-analyses. AMN is appointed as a research-assistant and will execute the study. All collaborators are considered as co-authors as they have significantly contributed to developing this research, obtaining the data, and writing the paper.

## Pre-publication history

The pre-publication history for this paper can be accessed here:

http://www.biomedcentral.com/1471-2431/11/28/prepub
